# Supplementary material for: Epithelial and Mesenchymal-like Pancreatic Cancer Cells Exhibit Different Stem Cell Phenotypes Associated with Different Metastatic Propensities
Source: Cancers (Basel). 2024 Feb 6;16(4):686. doi: 10.3390/cancers16040686 (PMC10886860; doi:10.3390/cancers16040686)
Supplement: Supplementary file 1 [file cancers-16-00686-s001.zip › Legends to Supplementary Figures_LMP.pdf]

Supplementary Figures

Supplementary Figure S1 Transcriptomic analysis of Panc1 and Panc89 cell variants revealed differences in their pathways activities. Comparative Gene Set Enrichment Analysis (GSEA) on the differential effect sizes of the reactome pathways in A) parental Panc1 and Panc89 cells, B) Panc1 Holo- and Paraclone cells and C) Panc89 Holo- and Paraclone cells. GSEA on the shrunken log2 fold changes from apeglm (58) was performed using GAGE (v2.48.0) (59) against Hallmark, Reactome and Gene Ontology Biological Processes (GOBP) gene sets extracted from the msigdbr R package (v7.5.1). RNA sequencing-based subtyping of D) parental Panc1 and Panc89 cells as well as their derived E) Holo- and Paraclone cells according to the classical-like and basal-like PDAC cell subtypes of Moffit et al. (61). Data are presented from 3 independent replicates of each cell variant.

Supplementary Figure S2 Panc1 and Panc89 cell variants show marginal differences in their adhesion abilities. Adhesion abilities of parental Panc1 and Panc89 as well as Holo- and Paraclone cells to liver endothelial, lung endothelial and mesothelial cells were analyzed. 1×10^5/well endothelial or mesothelial cells were seeded in a 96-well plate and after 24 h, CellTracker Green stained tumor cells were seeded on top of endothelial and mesothelial cell layer. After 4 h, the percentage of adherent tumor cells was determined. Parametric data are shown as mean with SEM, non-parametric data are presented as median with interquartile range. Significances are indicated by asterisks: $p \leq 0.033$ = *. (Holo = Holoclone cells, Para = Paraclone cells, SEM = Standard error of means)

Supplementary Figure S3 Pan-Cytokeratin staining of Panc1 and Panc89 tumors and cyst formation in Panc89 Paraclone tumors. SCID beige mice were inoculated intrasplenically with either 1×10^4 Panc1 Holo- or Paraclone cells or Panc89 Holo- or Paraclone cells (10 mice/group). Resected tissues and tumor lesions were stained for A) Pan-cytokeratin. Scale bars in the representative images: left = 500 μm; right = 100 μm. B) Representative images of cyst formation in two different Panc89 Paraclone tumors. Scale bar = 200 μm.

Supplementary Figure S4 Immunohistochemical analysis of L1CAM and SOX2 expression in Panc89 Holoclone tumors. SCID beige mice were inoculated intrasplenically with either 1×10^4 Panc89 Holoclone cells (10 mice/group). Resected tumors were stained for L1CAM and SOX2. Data of the comparative analysis of L1CAM and SOX2 expression in all Panc89 Holoclone tumors (left) and of Panc89 Holoclone tumors exhibiting high L1CAM expression (right) are presented as median with range in violin plots. Significances are indicated by asterisks: $p \leq 0.033$ = *.

Supplementary Figure S5 Parental Panc1 cells exhibit higher gene expression of matrix metalloproteases compared to parental Panc89 cells. Gene expression of matrix metalloproteases (MMP-2 and MMP-9 was analyzed by RT-qPCR in parental Panc1 and Panc89 cells. MMP expression was normalized to the reference gene GAPDH and data are shown as mean with SD. Significances are indicated by asterisks: $p \leq 0.033$ = *, $p \leq 0.002$ = **. (MMP = matrix metalloproteases, GAPDH = glycerinaldehyde 3-phosphate dehydrogenase, SD = standard deviation)
