# Supplementary material for: Epithelial and Mesenchymal-like Pancreatic Cancer Cells Exhibit Different Stem Cell Phenotypes Associated with Different Metastatic Propensities
Source: Cancers (Basel). 2024 Feb 6;16(4):686. doi: 10.3390/cancers16040686 (PMC10886860; doi:10.3390/cancers16040686)
Supplement: Supplementary file 1 [file cancers-16-00686-s001.zip › Supplementary Figure S1.pdf]

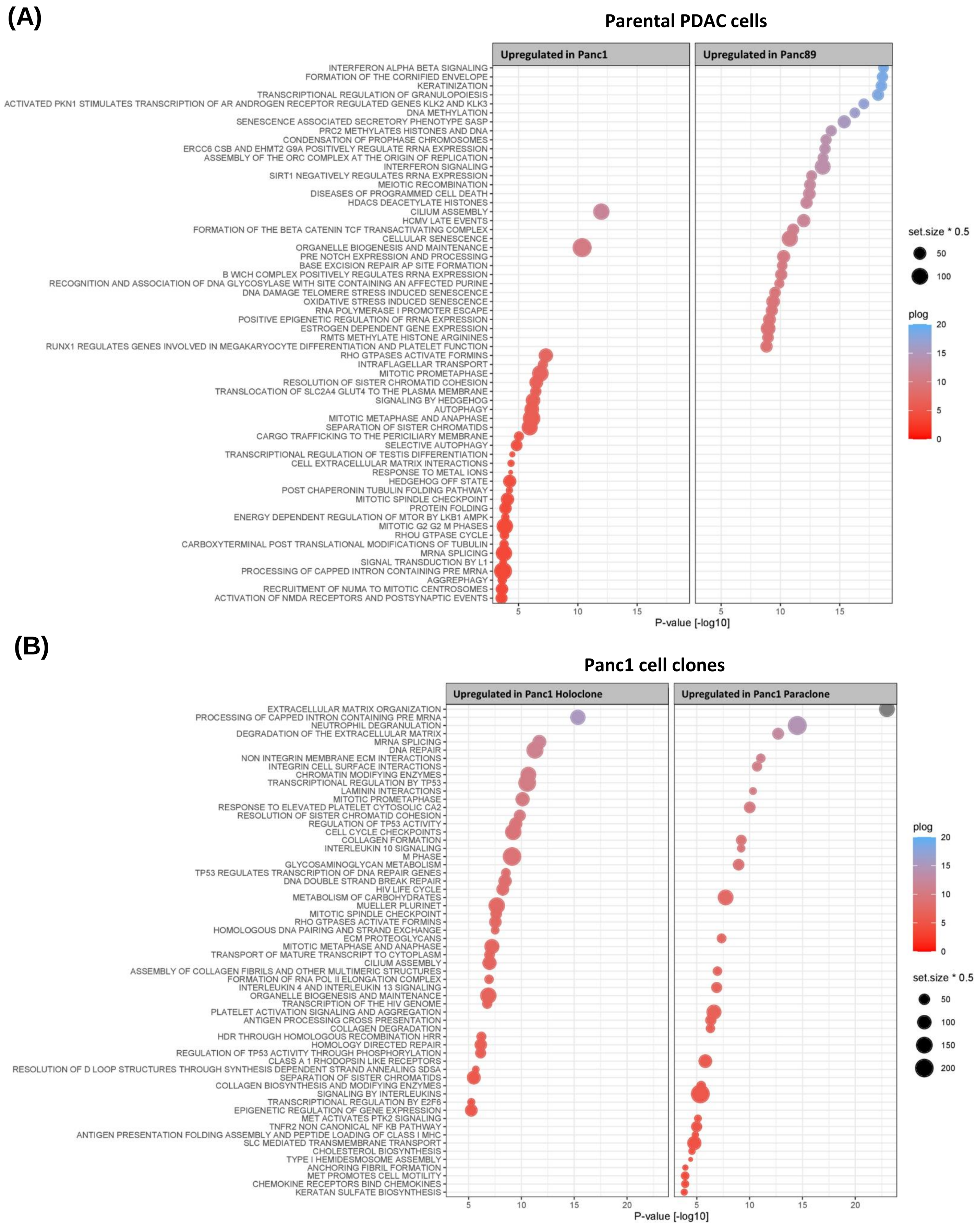

Supplementary Figure 1

(C)

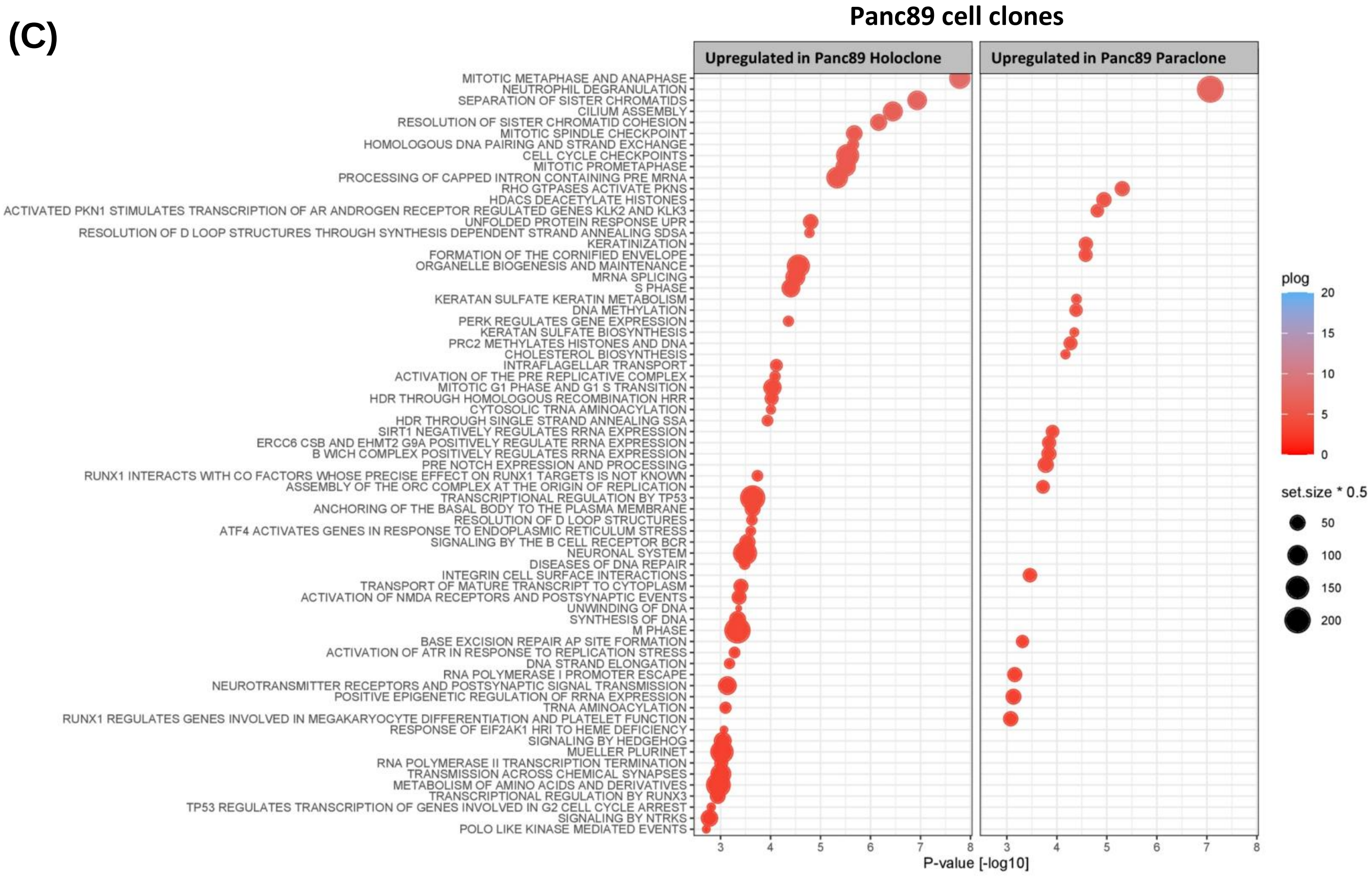

Supplementary Figure 1

(D)

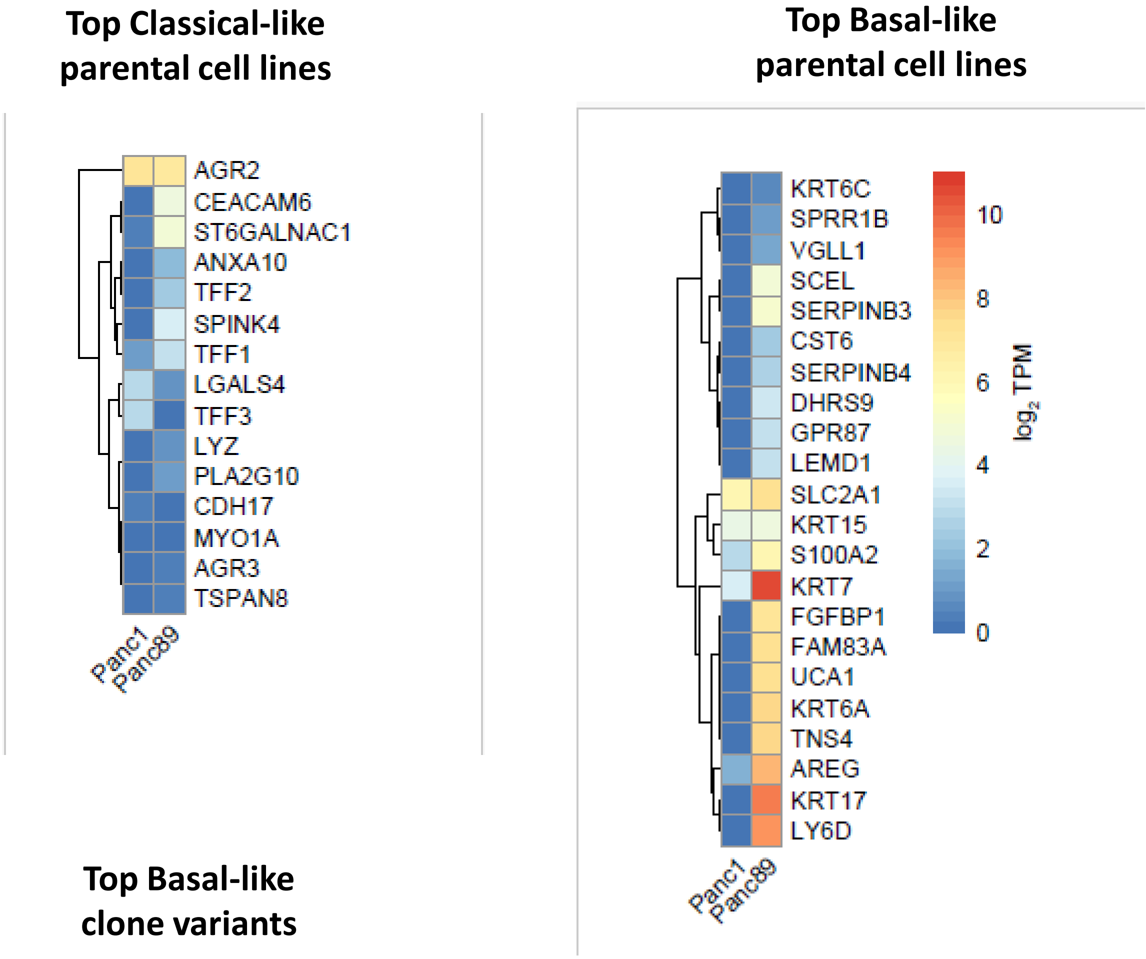

(E)

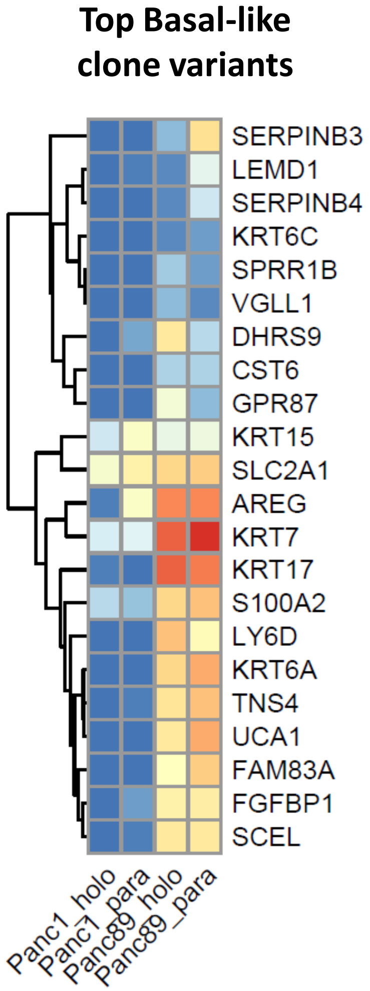

Supplementary Figure 1
