# Supplementary figures and images for: Epithelial and Mesenchymal-like Pancreatic Cancer Cells Exhibit Different Stem Cell Phenotypes Associated with Different Metastatic Propensities
Source: Cancers (Basel). 2024 Feb 6;16(4):686. doi: 10.3390/cancers16040686 (PMC10886860; doi:10.3390/cancers16040686)

### Adhesion to liver endothelium

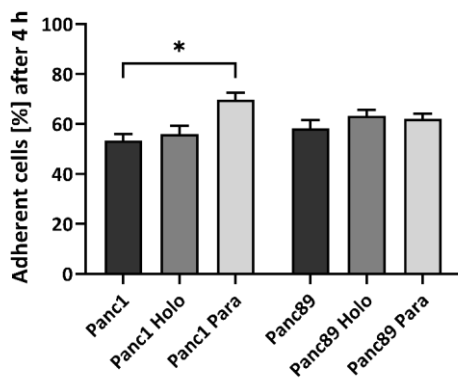

### Adhesion to lung endothelium

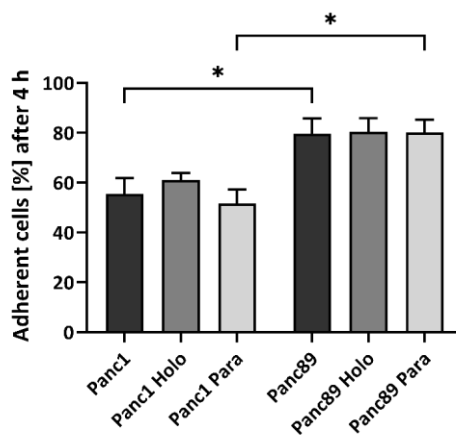

### Adhesion to mesothelial cells

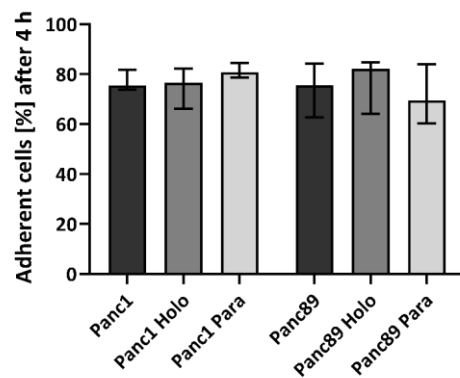

Supplement: Supplementary file 1 [file cancers-16-00686-s001.zip › Supplementary Figure S2.pdf]

(A)

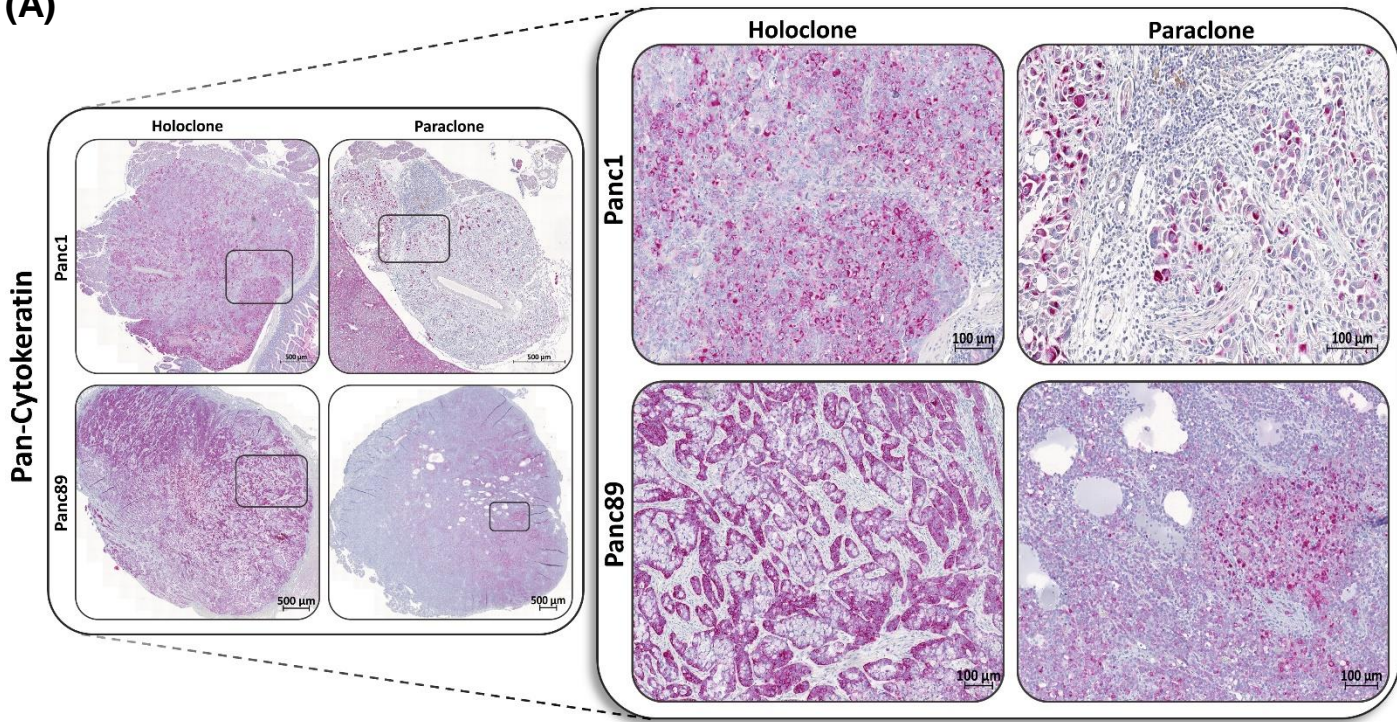

(B)

Cyst formation in Panc89 Paraclone tumors

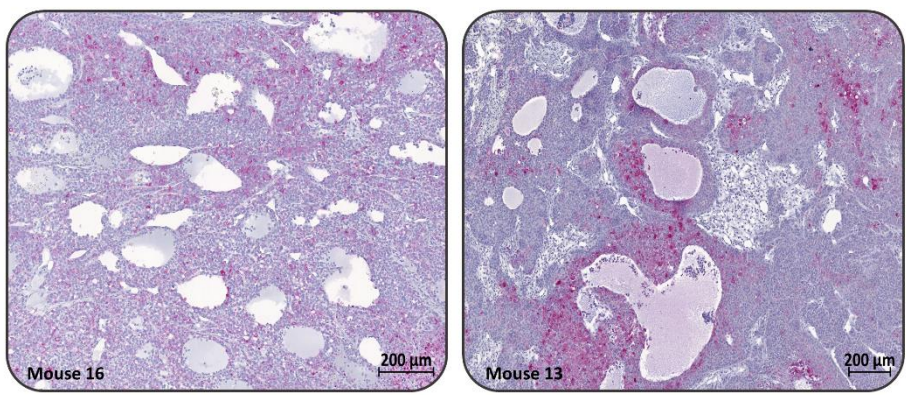

Supplement: Supplementary file 1 [file cancers-16-00686-s001.zip › Supplementary Figure S3.pdf]

Total Panc89 Holoclone tumors

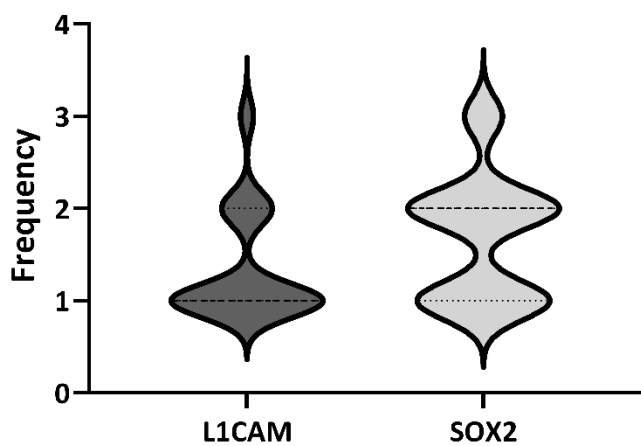

L1CAM high Panc89 Holoclone tumors

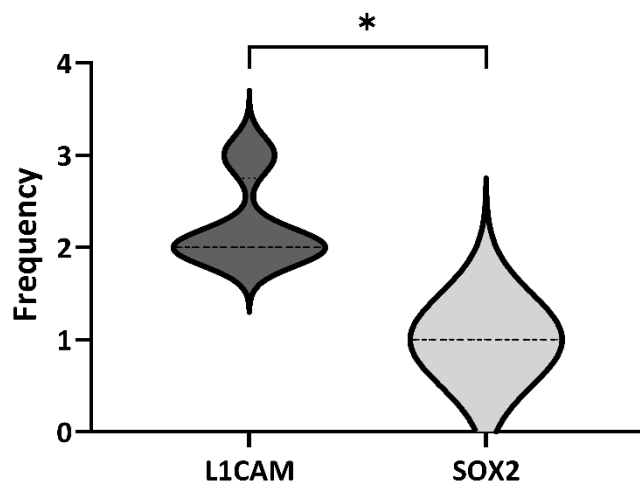

Supplement: Supplementary file 1 [file cancers-16-00686-s001.zip › Supplementary Figure S4.pdf]

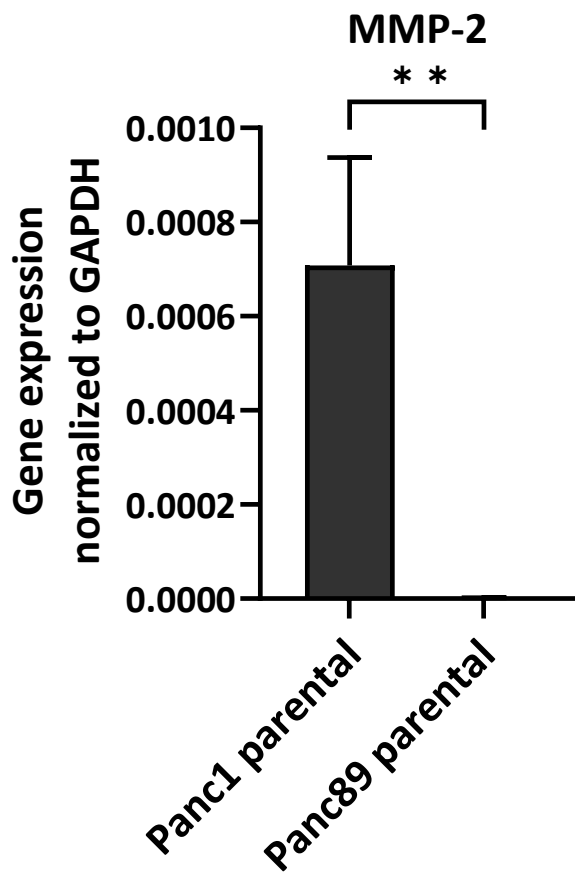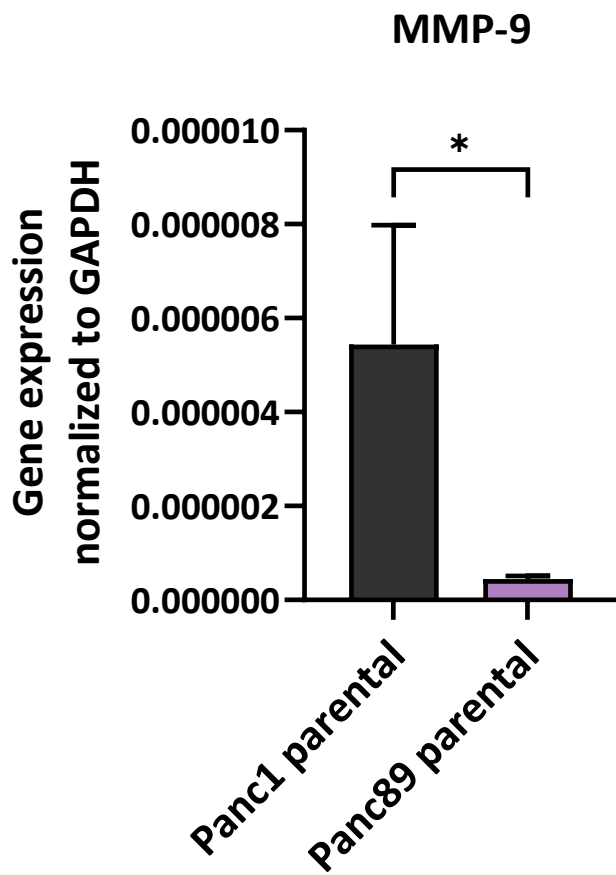

Supplement: Supplementary file 1 [file cancers-16-00686-s001.zip › Supplementary Figure S5.pdf]
